# Concurrent acute coronary and Takotsubo syndrome: two in one

**DOI:** 10.1007/s10554-023-02813-1

**Published:** 2023-03-13

**Authors:** M. Wagener, R. Twerenbold, Michael J. Zellweger, Philip Haaf

**Affiliations:** 1 Department of Cardiology and Cardiovascular Research Institute Basel (CRIB), University Hospital Basel, University of Basel, Basel, Switzerland; 2 Galway University Hospitals, Galway, Ireland; 3 University Center of Cardiovascular Science, University Heart and Vascular Center Hamburg, Clinic for Cardiology, University Medical Center Hamburg-Eppendorf, Hamburg, Germany

A-69-year-old woman with metabolic syndrome was referred for emergency coronary angiography with chest pain, signs of left ventricular decompensation, elevated high-sensitivity cardiac troponin T (3686 ng/l, norm < 14 ng/l) and inferolateral ST-segment elevations. Apart from the acute chest pain itself no further emotional or physical stress factors could be identified.

Coronary angiography revealed a 1-vessel coronary artery disease with a distally occluded right posterolateral branch (Panel 1a, 1b, Supplement Video S1) and elevated left ventricular end-diastolic pressure of 34 mmHg. Considering the tortuosity and small vessel diameter, the patient was treated conservatively with dual antiplatelet therapy. Echocardiography showed severely depressed ejection fraction with akinesia of the apical two thirds of the myocardium with basal hyperkinesia. Cardiovascular magnetic resonance (CMR) imaging showed evidence of a focal acute transmural inferolateral midventricular myocardial infarction (Panel 2a, 2b) as well as a myocardial oedema of the akinetic apical two thirds of the myocardium (Panel 3a, 3b and 4a-d, Supplement Video S2). The findings of the patient were interpreted as concurrent Takotsubo syndrome (TTS) and acute myocardial infarction.

Two months later, left-ventricular global systolic function completely normalized, myocardial oedema as well as apical ballooning resolved (Supplement Panel S1a-b, S2a-d, Supplement Video S3). Furthermore, wall thinning with dyskinesia developed of the transmurally infarcted inferolateral midventricular segment (Supplement Panel S3a-b) (Fig. 1).

**Fig. 1:**
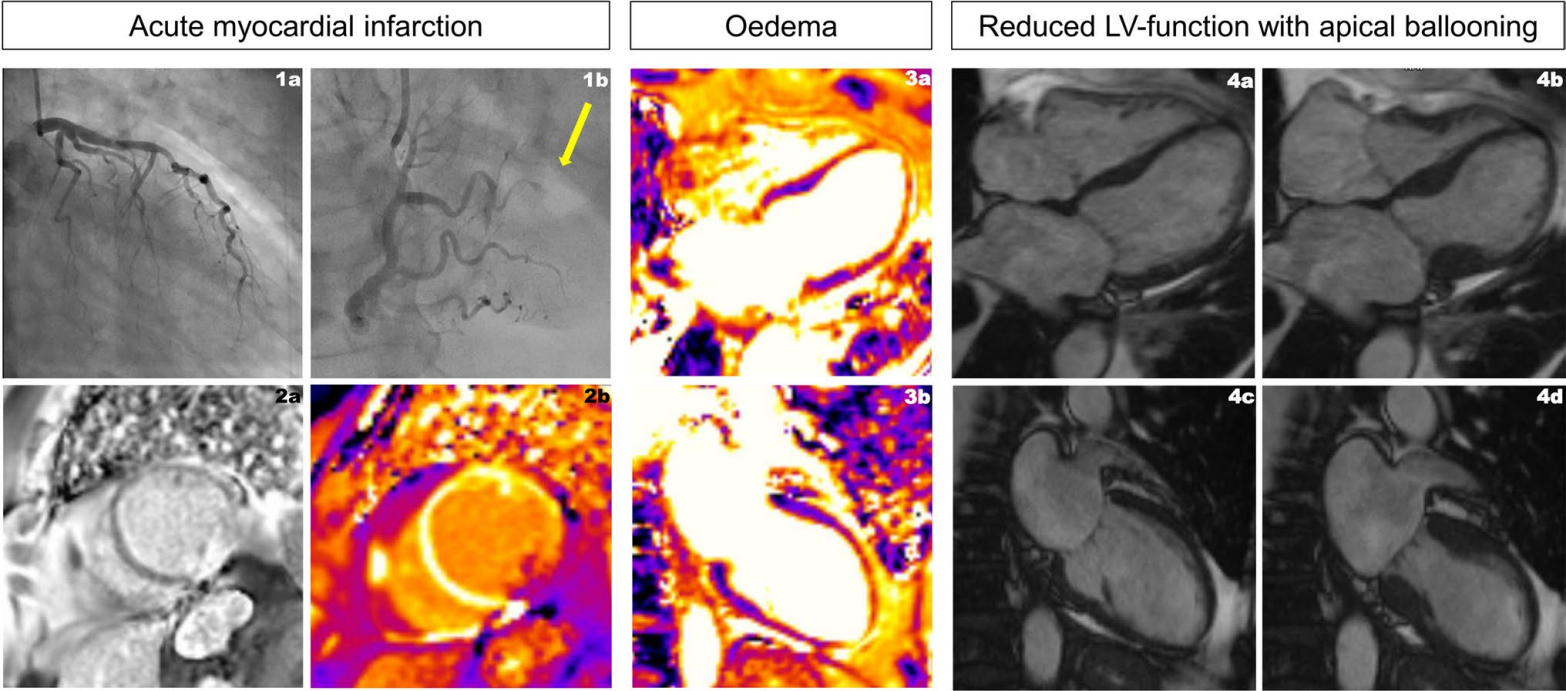
1a – RAO caudal view of the left anterior desending and circumflex artery; 1b – RAO cranial view of the right coronary artery with occluded distal right posterolateral branch (yellow arrow); 2a/b – Cardiovascular Magnetic Resonance (CMR) imaging with evidence of a focal acute transmural inferolateral midventricular myocardial infarction (2a: Late Gadolinium Enhancement; 2b: T1 post-contrast Map); 3a and 3b – CMR T2 map showing myocardial oedema of the apical two thirds of the myocardium; 4a–d –four chamber view cine images in diastole (4a) and apical ballooning in systole (4b); two chamber view cine images in diastole (4c) with apical ballooning in systole (4d)

## Discussion

Acute coronary syndrome (ACS) is still widely regarded as an exclusion criterion of TTS and discrimination between them can sometimes be challenging. Although, concomitant coronary artery disease in TTS is known to be present in around 10–29% of cases, patients with concurrent TTS and acute obstructive CAD are often misdiagnosed as classical ACS [1] since presence of ACS is still widely regarded as an exclusion criterion for TTS.

A multimodality imaging approach including CMR should be considered when coronary angiography findings do not match with ventriculography or echocardiography. The presence of myocardial infarction should not exclude TTS per se but ACS may occasionally trigger - rather than exclude TTS, as recently outlined in the latest consensus paper on TTS [2].

## Supplementary Information

Below is the link to the electronic supplementary material.

Supplementary material 1 (JPG 978 kb)

Supplementary file2 (M4V 720 kb)

Supplementary file3 (MP4 5836 kb)

Supplementary file4 (MP4 5417 kb)
