# Supplementary figures and images for: Concurrent acute coronary and Takotsubo syndrome: two in one
Source: Int J Cardiovasc Imaging. 2023 Mar 13;39(6):1179–80. doi: 10.1007/s10554-023-02813-1 (PMC10220098; doi:10.1007/s10554-023-02813-1)

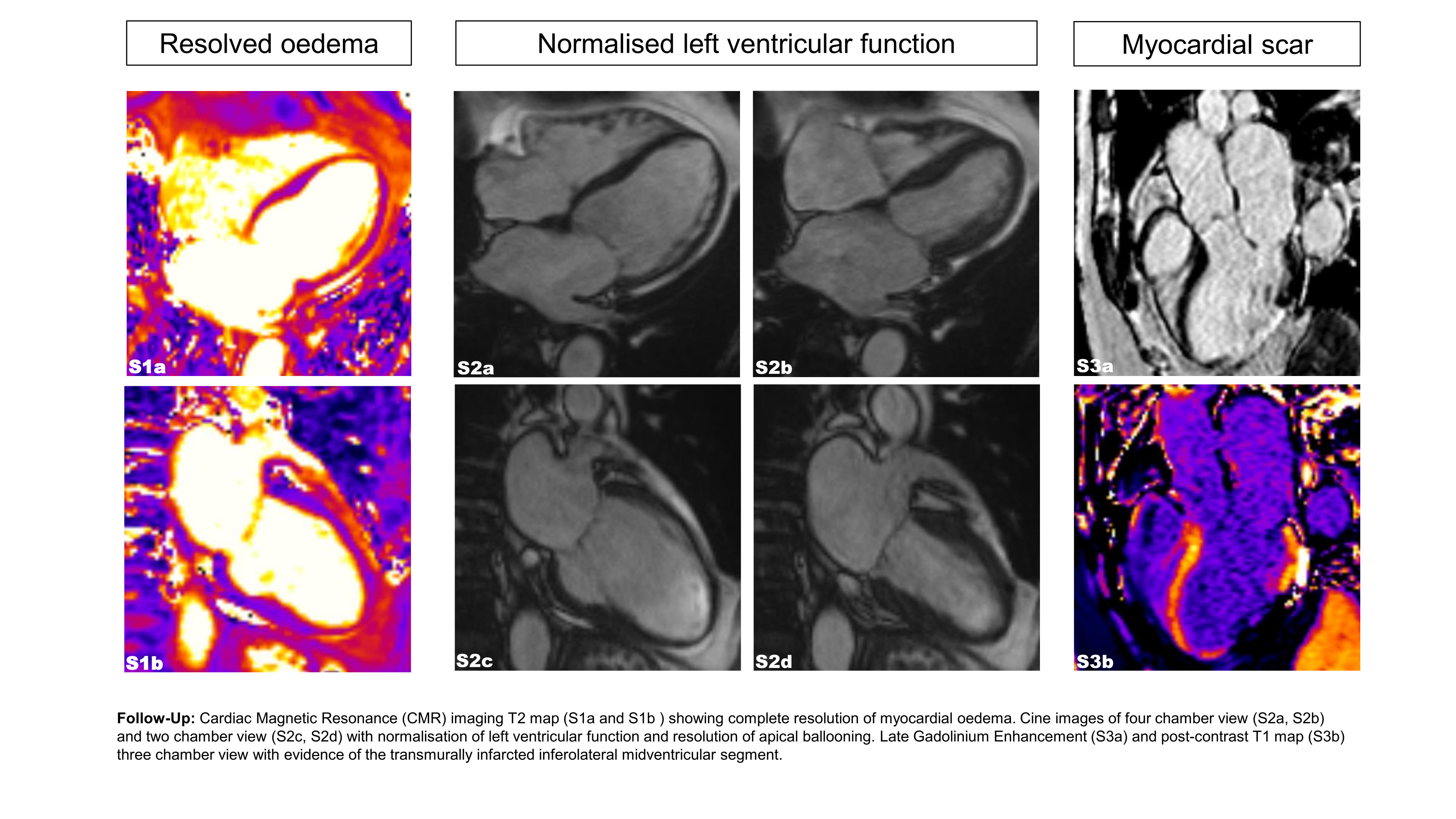

Supplement: Supplementary file 1 — Supplementary material 1 (JPG 978 kb) [file 10554_2023_2813_MOESM1_ESM.jpg]
